# Supplementary material for: The effect of carbon monoxide on meiotic maturation of porcine oocytes
Source: PeerJ. 2021 Mar 23;9:e10636. doi: 10.7717/peerj.10636 (PMC7996072; doi:10.7717/peerj.10636)
Supplement: Supplemental Information 7 — Oocytes were cultivated for 48 h in a modified M199 medium supplemented with iCORM-2 (100 µM) or iCORM-A1 (100 µM). Control groups of oocytes were cultivated in a modified M199 medium containing DMSO or distilled H2O. DMSO or distilled H2O was added in an equivalent volume as iCORM-2 or iCORM-A1. The significant difference in the ratio of oocytes between the appropriate control and iCORM group are indicated with different superscripts a b (P < 0.05). GV (germinal vesicle), MI (metaphase I), MII (metaphase II), Deg (abnormal oocytes). [file peerj-09-10636-s007.docx]

| Stage | Control group | | iCORM group | |
| --- | --- | --- | --- | --- |
|  | C (DMSO) | C (H_2_O) | iCORM-2 | iCORM-A1 |
| GV | 5.9 ± 1.3 ^a^ | 4.4 ± 1.3 ^a^ | 5.4 ± 1.4 ^a^ | 6.4 ± 1.9 ^a^ |
| MI | 7.3 ± 0.9 ^a^ | 9.5 ± 1.6 ^a^ | 7.8 ± 1.9 ^a^ | 7.0 ± 0.9 ^a^ |
| MII | 84.3 ± 0.8 ^a^ | 84.7 ± 2.0 ^a^ | 83.9 ± 1.8 ^a^ | 80.8 ± 2.2 ^a^ |
| Deg | 2.4 ± 1.0 ^a^ | 1.3 ± 1.6 ^a^ | 2.4 ± 1.9 ^a^ | 4.6 ± 1.8 ^a^ |

Supplementary Table S1:

**The effect of inactive CORM-2 (iCORM-2) and inactive CORM-A1 (iCORM-A1) on meiotic maturation of porcine oocytes.** Oocytes were cultivated for 48 hours in modified M199 medium supplemented with iCORM-2 (100 µM) or iCORM-A1 (100 µM). Control groups of oocytes were cultivated in modified M199 medium containing DMSO or distilled H2O. DMSO or distilled H2O were added in an equivalent volume as iCORM-2 or iCORM-A1. Significant difference in the ratio of oocytes between appropriate control and iCORM group are indicated with different superscripts ^a b^ (P < 0.05). GV (germinal vesicle), MI (metaphase I), MII (metaphase II), Deg (abnormal oocytes).
