# Supplementary material for: The effect of carbon monoxide on meiotic maturation of porcine oocytes
Source: PeerJ. 2021 Mar 23;9:e10636. doi: 10.7717/peerj.10636 (PMC7996072; doi:10.7717/peerj.10636)
Supplement: Supplemental Information 8 — These specific primers were used to analyze the expression levels of HO-1 and HO-2 mRNA during the meiotic maturation of porcine oocytes. [file peerj-09-10636-s008.docx]

| Gene | Forward primers 5´-3´ | Reverse primers 5´-3´ | TagMan probe 5´-3´ | Gene accession number |
| --- | --- | --- | --- | --- |
| HO-1 | TCCCAGGTGCCGCTCAT | CCGTTGCCACCAGAAAGC | CGATGGGTCCTGACACT | NM_001004027.1 |
| HO-2 | TGAGAAAACAAGCACCCAATAAAA | CCGCAGACAAGATGCTGACA | CAGATGCTAAAGCCTG | NM_001244412.1 |

Supplementary Table S2 The sequences of the specific primers used for RT-PCR Amplification of heme oxygenase-1 and heme oxygenase-2. The sequences of primers were based on the knowledge of HO-1 and HO-2 gene sequence.
